# SHP2 inhibition by SHP099 attenuates IL-6–driven osteoclastogenesis in growth plate injury

**DOI:** 10.3389/fimmu.2025.1659230

**Published:** 2025-08-15

**Authors:** Qin Zhang, Ning Li, Zhen-Zhen Dai, Xiao-Man Liu, Jing Ding, Lin Sha, Hai Li

**Affiliations:** ^1^ 1Department of Pediatric Orthopedics, Xin Hua Hospital Affiliated to Shanghai Jiao Tong University School of Medicine, Shanghai, China; ^2^ Shanghai Jiao Tong University School of Medicine, Shanghai, China; ^3^ Department of Neurosurgery, The First Affiliated Hospital of Bengbu Medical University, Anhui, China; ^4^ Graduate School of The Bengbu Medical University, Anhui, China; ^5^ Department of Gastroenterology, Xin Hua Hospital Affiliated to Shanghai Jiao Tong University School of Medicine, Shanghai, China; ^6^ Department of Pediatric Orthopedics, Shanghai Children’s Hospital Affiliated to Shanghai Jiao Tong University School of Medicine, Shanghai, China

**Keywords:** SHP2, SHP099, IL-6, osteoclasts, growth plate injury

## Abstract

**Introduction:**

Disruption of growth plate cartilage often leads to severe bone growth defects in children, necessitating novel therapeutic strategies. Following growth plate injury, an inflammatory response is rapidly initiated, resulting in the release of pro-inflammatory cytokines such as IL-6 into the injured tissue, which subsequently induce and enhance osteoclast generation and differentiation. This study investigates the role of SHP2 in regulating IL-6-driven osteoclastogenesis during growth plate injury repair.

**Methods:**

Tibial drill-hole injuries were induced in C57BL/6 mice (n=9), with SHP099 (30 mg/kg, intra-articular) administered to intervention groups and tissues were harvested for qPCR/histology. RAW 264.7 cells were treated with RANKL (100 ng/ml) ± IL-6 (100 ng/ml) ± SHP099 (15 µM). Osteoclast differentiation, expression level of pro-inflammatory cytokines and the associated signaling pathway were assessed via TRAP staining, Western blot, qPCR and ELISA.

**Results:**

SHP2/PTPN11, osteoclast markers (CTSK/OSCAR) and pro-inflammatory cytokines (IL-6, IL-1β, TNF-α) was upregulated and could be inhibited by SHP099 at injury sites. IL-6 enhanced p-SHP2/p-TAK1 expression, osteoclastogenesis and inflammatory response in vitro, while SHP099 effectively reduced osteoclast numbers, downregulating CTSK/OSCAR and pro-inflammatory cytokines (IL-6, IL-1β, TNF-α). Furthermore, the NF-κB pathway remained unaffected by SHP099, indicating a distinct signaling mechanism through which SHP2 regulates osteoclastogenesis.

**Discussion:**

Our findings underscore the pivotal role of SHP2 as a downstream signaling molecule of IL-6 in mediating inflammatory responses during bone repair, suggesting that SHP2 inhibition may present a novel therapeutic approach to prevent pathological bone remodeling and enhance recovery following growth plate injuries. Future investigations should focus on the translational potential of SHP2 inhibitors in pediatric orthopedics.

## 1 Introduction

Bone fractures are unfortunately common among children and adolescents, with approximately 50% of these injuries occurring in this population (1). Notably, 20% of all fractures occur at the growth plate, the weakest part of long bones (2). These fractures are often associated with serious post-injury complications, such as bone dysrepair characterized by the formation of local bone bridges and/or growth defects (3). Current surgical methods to correct these bone disorders can be invasive and sometimes ineffective, highlighting the urgent need to explore and gain a deeper mechanistic understanding of the processes underlying impaired healing to develop preventive management strategies.

A substantial body of research over the past two decades has identified four main phases of the repair response following injury to the epiphyseal plate: the inflammatory, fibrogenic, osteogenic, and remodeling stages (4). Osteoblasts, osteoclasts, and various cytokines play indispensable roles in the post-injury repair process. The inflammatory response is rapidly activated, leading to the production and secretion of significant inflammatory factors, such as interleukin-6 (IL-6) (5), interleukin-1β (IL-1β) (6) and Tumor Necrosis Factor alpha (TNF-α) (4). Numerous studies have demonstrated that these cytokines promote the proliferation, differentiation, and maturation of osteoclasts, particularly IL-6 (7–9). Osteoclasts, as key regulators of bone remodeling, significantly contribute to the formation and stabilization of bone bridges, thereby physically impeding the growth of the epiphyseal plate. This process represents a primary pathological basis for limb length discrepancies and angular deformities.

Osteoclasts express high levels of several members of the protein tyrosine phosphatase (PTP) superfamily, notably PTPN11 (10), which encodes tyrosine-protein phosphatase non-receptor type 11 (SHP2). SHP2 functions downstream of various receptor and cytoplasmic protein tyrosine kinases, playing a critical role in signal transduction from the cell surface to the nucleus, which is essential for osteoclastogenesis (10, 11). Furthermore, the binding of IL-6 to its receptor (IL-6R) activates one of the major intracellular signaling pathways involving SHP2 (12). Evidence from a growing body of research indicates that genetic deletion of PTPN11 results in reduced osteoclast numbers and function following SHP2 inhibition (11, 13). SHP099 (IC_50_ = 71 nmol/L), an allosteric inhibitor of SHP2, binds concurrently to the interfaces of the protein tyrosine phosphatase domains, as well as the N-terminal and C-terminal SH2 domains (14). This compound has been investigated in various diseases, including osteoarthritis (15). Investigating the role of SHP2 in the differentiation and proliferation of osteoclasts during the repair process following growth plate injury, as well as the potential application of its allosteric inhibitor, may provide new therapeutic avenues at the cellular and molecular levels for alleviating and even preventing the formation of bone bridges. In our work, we found that SHP2, activated by IL-6 in osteoclasts, is crucial for promoting osteoclastogenesis through the TAK1/NF-κB (p65) signaling pathway. Targeting SHP2 with SHP099 may theoretically offer new insights into preventing bone bridge formation after growth plate injury.

## 2 Materials and methods

### 2.1 Animals and treatment

All animal experiments were conducted in accordance with the guidelines of the Animal Care and Use Committee of Xinhua Hospital affiliated with Shanghai Jiao Tong University. Nine male C57BL/6 mice, aged 3–4 weeks, were procured from Shanghai Jihui Laboratory Animal Care Co. and randomly allocated into three cages (three mice per cage). Two cages underwent growth plate injury surgery, where a drill hole was created in the proximal tibia bilaterally under anesthesia, as previously described (16). The specific intraoperative measure is to use the needle of a 1ml sterile syringe (with a diameter of approx. 0.45mm) to drill a hole vertically down the tibia instead of an electric drill. SHP099 was administered intraarticularly into the right knee at a dosage of 30 mg/kg. Three non-operated mice were euthanized to serve as the control group (CTRL), yielding six left knees for the growth plate injury group (GPI) and six right knees for the GPI+SHP099 group. Tissues were harvested approximately 10 days post-surgery, with knees cryopreserved in liquid nitrogen or fixed in 4% paraformaldehyde for subsequent analyses.

The number of biological replicates was determined *a priori* by power analysis using G Power 3.1 software. Based on preliminary experiments measuring osteoclast marker (CTSK) expression in GPI vs CTRL groups, we observed an effect size of Cohen’s *d* = 1.5 (mean difference = 2.3-fold, SD = 0.52). For α = 0.05 and power (1-β) = 0.8, a minimum sample size of n = 5 per group was required for Student’s t-test. To account for potential technical variability, we used n ≥ 6 for qPCR and n = 3 for histological assays (consistent with published standards in bone injury models) (17, 18).

### 2.2 Cell culture and treatment

RAW 264.7 cells were maintained in Dulbecco’s modified Eagle medium (DMEM) supplemented with 10% fetal bovine serum (FBS) and 1% penicillin-streptomycin, in a humidified atmosphere of 5% CO2 at 37°C. Cells between the tenth and fifteenth passages were seeded in 48-well and 6-well plates at densities of approximately 1 × 10^4 and 8 × 10^4 cells per well, respectively. Induction media (α-MEM) containing 100 ng/ml RANKL (19), 100 ng/ml IL-6, 15 μM SHP099, and 7.5 μM BAY 11–7082 were administered, with media changed every other day. Cells were harvested for subsequent experiments after a 5 to 6-day incubation period. All details of reagents were showed in **Supplementary Table 1**.

### 2.3 Western blotting

Cells (n=3) were lysed using high-intensity RIPA lysis buffer (Beyotime Biotechnology) supplemented with 1 mM PMSF, phosphatase inhibitor, and protease inhibitor. Protein concentrations were quantified using the BCA Assay Kit (Beyotime Biotechnology). Extracted proteins (10-20 µg) were resolved by SDS-PAGE and transferred to polyvinylidene difluoride membranes. After blocking with Quick Block solution (Beyotime Biotechnology) for 15–30 minutes, membranes were incubated overnight at 4°C with primary antibodies diluted 1:1000-1:2000. Horseradish peroxidase-conjugated secondary antibodies (1:5000) were applied, and protein detection was achieved using enhanced chemiluminescence (Bioagri). All details of antibodies and reagents were showed in **Supplementary Table 1**. Signal intensities were quantified using NIH ImageJ software.

### 2.4 Quantitative real-time polymerase chain reaction

RNA was extracted from cells (n=6) using RNA/DNA Isolation Kit. qPCR reactions were performed in a 10 µL volume using the SYBR Green q-PCR Kit (D7262, 2X, Low ROX) with specific primers sourced from Beyotime Biotechnology. All primers’ details were showed in **Supplementary Table 1**.

### 2.5 Enzyme-linked immunosorbent assays

Cell supernatants were collected after centrifugation (4°C, 3000g × 10 min) for analysis. Concentrations of IL-1β, TNF-α, and IL-6 in cell supernatants (n=3) were quantified according to the manufacturer’s instructions using an ELISA development kit (EK201B, EK282, EK206, Multi Sciences). Added standards, samples, and detection antibody sequentially within 15 minutes. Sealed the plate and incubated with oscillation (100–300 rpm) at 25°C ±3°C for 2 hours. Performed six stringent wash cycles (300 μL/wash, thorough aspiration/patting) after primary and secondary incubations. Added substrate, incubated protected from light (5–30 min, 25°C ±3°C), then stopped the reaction with acid for spectrophotometric quantification (color change: blue → yellow). Absorbance was measured at 450 nm and 570 nm using a Microplate Reader (Thermo Scientific).

### 2.6 Tartrate-resistant acid phosphatase staining assay

TRAP activity was assessed using a TRAP Stain Kit (Solarbio, Cat: G1492) following the manufacturer’s guidelines. Fixed cells in pre-chilled (2–8°C) TRAP fixative for 30–60 s, rinsed, then incubated in TRAP working solution (37°C, humidified chamber, 45–60 min). Counterstained with methyl green (2–3 min), rinsed with distilled water, and observed hydrated under 1x PBS with the light microscopy. The criteria for interpretation: TRAP^+^ cells with ≥3 nuclei are counted as osteoclasts.

### 2.7 Hematoxylin and eosin staining

Formalin-fixed, paraffin-embedded samples were sectioned at 6 µm and stained with hematoxylin and eosin to visualize tissue architecture.

### 2.8 CCK8 assay

Cells were plated in 96-well plates at a density of 2.5 × 10^4 cells/well and cultured for 24 hours. Following this, cells were treated with varying concentrations of SHP099 and BAY for 48 hours. CCK-8 reagent (BB-4202-500T; BestBio, Nanjing, China) was added (10 µL/well), and the plates were incubated for an additional 2–4 hours. Absorbance at 450 nm was measured using a microplate reader (Stat Fax-4200, USA), and cell survival rates were calculated using the formula: cell survival rate (%) = [(administration group A – negative control group A)/(non-administration group A – negative control group A)] × 100%.

### 2.9 Data analysis

Statistical analysis was performed using unpaired Student’s t-test for two-group comparisons, while one-way analysis of variance (ANOVA) was employed for comparisons across three to five groups. The normality of the data was tested by Shapiro-Wilk (*P* > 0.05); The homogeneity of variance was tested by Levene’s test (*P* > 0.10); Multiple comparisons are corrected by Tukey’s method. All data were visualized using GraphPad Prism 10 software (GraphPad Software, CA, USA). Results are presented as mean ± standard error of mean (SEM), with P < 0.05 considered statistically significant (*P < 0.05, **P < 0.01, ***P < 0.001, ****P < 0.0001), and “ns” indicating non-significance. All experiments were independently verified by multiple researchers.

## 3 Results

### 3.1 Osteoclasts and the gene expression level of PTPN11 increased after growth plate injured in mice

**Figure 1:**
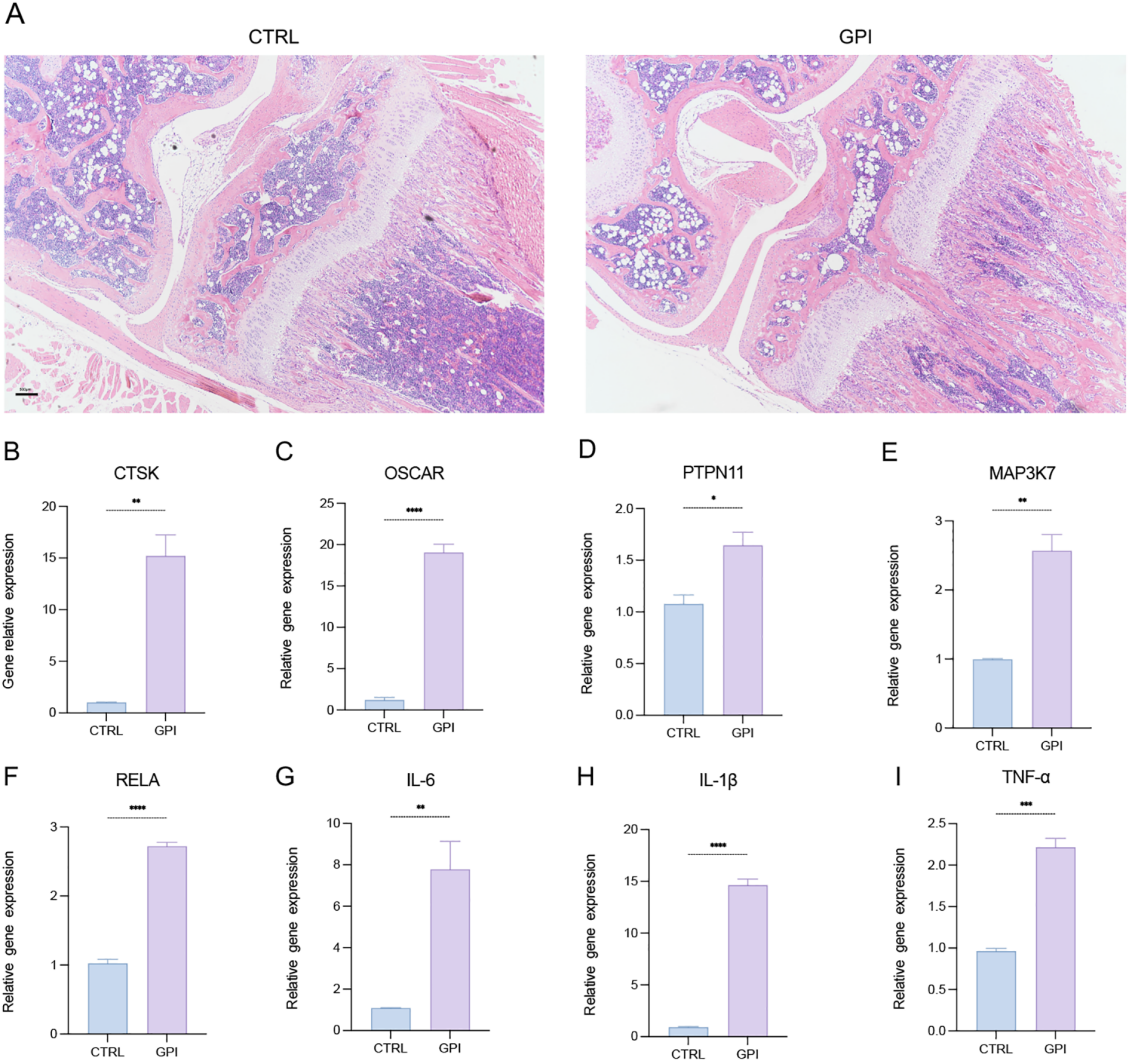
Osteoclasts and the gene expression level of PTPN11 increased after growth plate injury in mice. **(A)** Hematoxylin and Eosin (HE) staining of the mice knee joint. (n=3). 4X. **(B-I)** qPCR analysis of mRNA of Cathepsin K (CTSK), tyrosine-protein phosphatase non-receptor type 11 (PTPN11), Mitogen-Activated Protein Kinase Kinase Kinase 7 (MAP3K7), NF-Kappa-B Transcription Factor P65 (RELA), Osteoclast Associated Ig-Like Receptor (OSCAR), interleukin-6 (IL-6), IL-1β and tumor necrosis factor α (TNF-α). (n ≥ 6). Student’s t-test. *P < 0.05, **P < 0.01, ***P < 0.001, ****P < 0.0001. All data are shown as the mean SEM.

**Figure 2:**
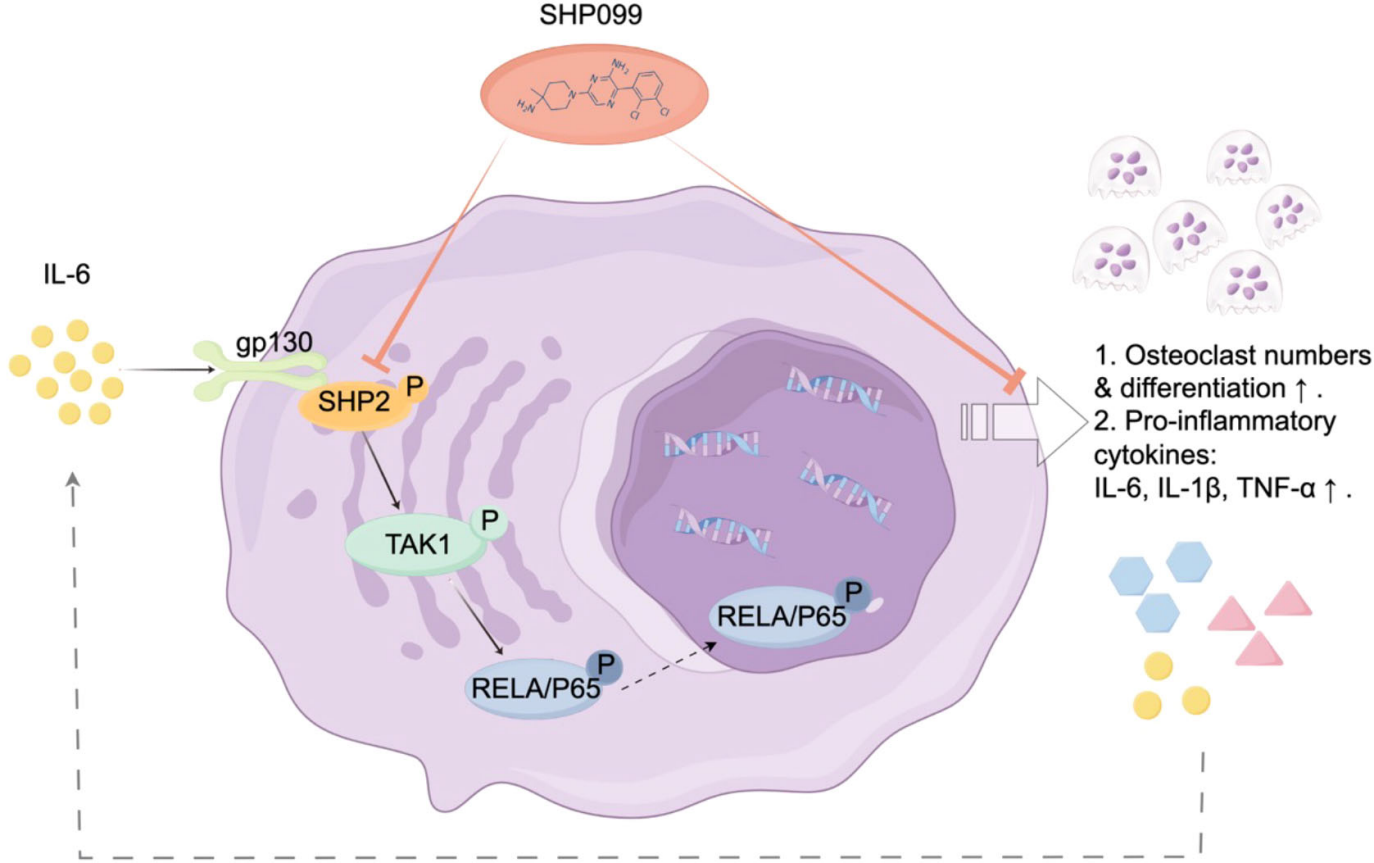
Schematic illustration of the mechanism of SHP2 in IL-6-driven osteoclastogenesis. Following injury, interleukin-6 (IL-6) binds to its receptor gp130, triggering the phosphorylation and activation of SHP2. This activation enhances the expression of TAK1 and the osteoclastogenic transcription factor NF-κB, leading to increased osteoclast differentiation and function. Treatment with SHP099, a specific inhibitor of SHP2, significantly reduces osteoclast numbers and downregulates key osteoclast markers, including cathepsin K (CTSK) and OSCAR, as well as pro-inflammatory cytokines such as IL-6, IL-1β, and TNF-α. This signaling pathway underscores the therapeutic potential of SHP2 inhibition in mitigating IL-6-driven osteoclastogenesis and abnormal bone remodeling following growth plate injuries.

**Figure 3:**
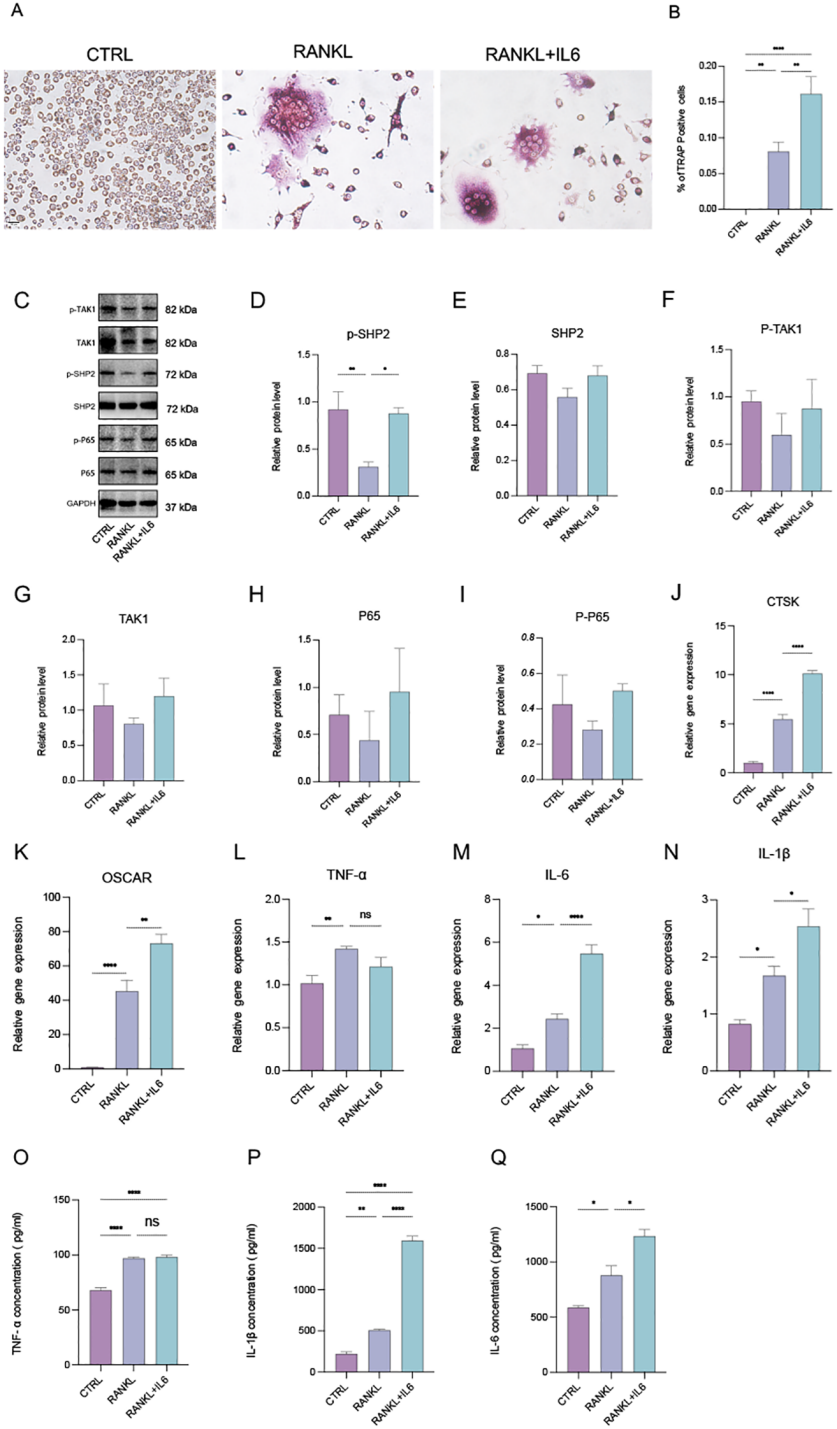
IL-6 promotes phosphorylated SHP2 up-expression and the osteoclastogenesis *in vitro*. **(A)** Multinucleated cells were viewed by light microscopy and stained by TRAP after induced by RANKL (100ng/ml) with or without IL-6 (100ng/mL) for 5 days. 40X. **(B)** Multinucleated osteoclastic cells were quantified by the amount of TRAP‐positive multinucleated cells (n=6). **(C)** Western blot analysis of src-homology 2-containing protein tyrosine phosphatase 2 (SHP2), phosphate SHP2 (p-SHP2), NF-κB/p65 (P65), phosphate p65 (p-P65), Mitogen-activated protein kinase kinase kinase 7 (TAK1), phosphate TAK1 (p-TAK1) in RAW264.7 after RANKL stimulation with or without IL-6 treatment for 5 days. **(D-I)** semiquantitative analysis of Western blot. (n ≥ 3). **(J-N)** qPCR analysis of mRNA of interleukin-1β (IL-1β), IL-6, tumor necrosis factor α (TNF-α), Cathepsin K (CTSK) and Osteoclast Associated Ig-Like Receptor (OSCAR). (n ≥ 6). **(O-Q)** Enzyme-linked immunosorbent assay (ELISA) results of TNF-α, IL-1β and IL-6 secretion. (n=3). One-way ANOVA test. *P < 0.05, **P < 0.01, ***P < 0.001, ****P < 0.0001, ns represents no significance. All data are shown as the mean SEM.

To investigate the pathological changes following growth plate injury (GPI), we performed surgery on mice and harvested their knee joints 10 days post-operation. The harvested tissues were divided equally, with half being prepared for paraffin embedding and the other half for RNA extraction. The paraffin-embedded sections were subsequently stained with hematoxylin and eosin (HE) (**Figure 1A**), confirming the success of the surgical procedures. We assessed the expression levels of marker genes associated with osteoclasts, pro-inflammatory cytokines, and various signaling pathways in both the control (CTRL) and GPI groups using quantitative reverse transcription polymerase chain reaction (RT-qPCR). Notably, the expression of osteoclast markers CTSK and OSCAR was significantly elevated in the GPI group (**Figures 1B, C**), indicating an increase in both the number and activity of osteoclasts at the injury site following growth plate injury. This finding highlights the activation of bone resorption processes during injury repair and bone remodeling. Additionally, we examined the expression levels of pro-inflammatory cytokines IL-6, IL-1β, and TNF-α, which were also found to be upregulated in the GPI group (**Figures 1G–I**). Furthermore, the expression of genes involved in relevant signaling pathways, particularly PTPN11, MAP3K7, and RELA, was significantly increased in the GPI group (**Figures 1D–F**). Concomitant upregulation of PTPN11 (SHP2), MAP3K7 (TAK1), and RELA (p65) post-injury suggested activation of a hierarchical signaling cascade: IL-6 → SHP2 → TAK1 → NF-κB (**Figure 2**). This axis aligns with known roles of SHP2 in transducing IL-6 signals (12), TAK1 in osteoclastogenesis (validated in **Figure 3**), and NF-κB in inflammatory bone remodeling (20). These findings suggest that these genes may play a crucial role in regulating osteoclastogenesis following growth plate injury.

### 3.2 SHP099 inhibits osteoclastogenesis and inflammation response after growth plate injured in mice

**Figure 4:**
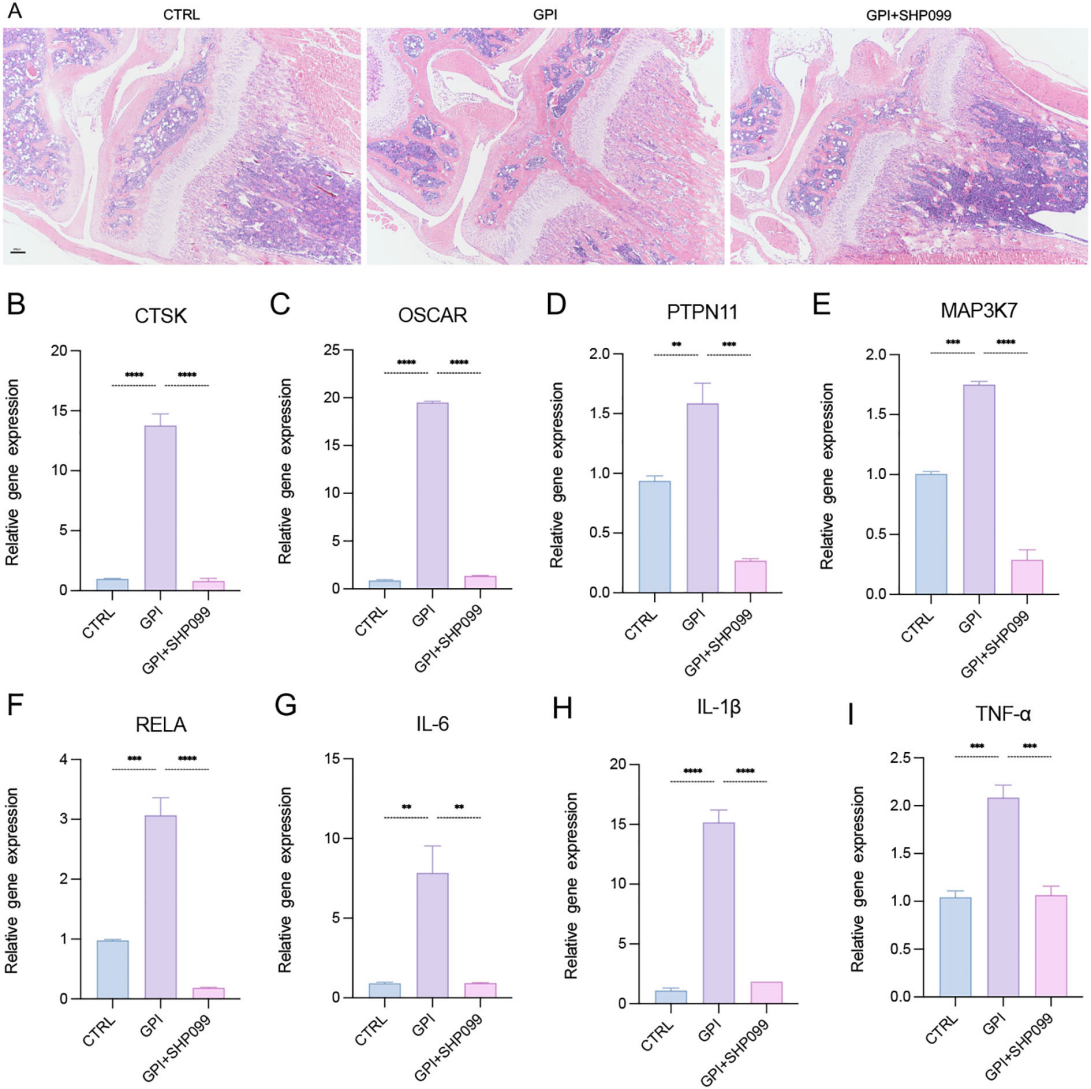
SHP099 inhibits the osteoclastogenesis and inflammation response after growth plate injury in mice. **(A)** Hematoxylin and Eosin (HE) staining of the mice knee joint. (n=3). 4X. **(B-I)** qPCR analysis of mRNA of Cathepsin K (CTSK), tyrosine-protein phosphatase non-receptor type 11 (PTPN11), Mitogen-Activated Protein Kinase Kinase Kinase 7 (MAP3K7), NF-Kappa-B Transcription Factor P65 (RELA), Osteoclast Associated Ig-Like Receptor (OSCAR), interleukin-6 (IL-6), IL-1β and tumor necrosis factor α (TNF-α). (n ≥ 6). One-way ANOVA test. **P < 0.01, ***P < 0.001, ****P < 0.0001. All data are shown as the mean SEM.

To evaluate the therapeutic effects of SHP099 on the inflammatory and osteoclastogenic responses following growth plate injury (GPI), SHP099 was administered via intra-articular injection into the knees of mice subjected to GPI. We conducted a comparative analysis among the control (CTRL), GPI, and GPI + SHP099 treatment groups. Histological examination using hematoxylin and eosin (HE) staining revealed no notable macroscopical differences in the knee joint tissues across the groups (**Figure 4A**), as osteogenic bridge formation required ≥14 days for histological manifestation or SHP099 likely exerted its therapeutic effect by preemptively suppressing osteoclastogenesis (evidenced by reduced CTSK/OSCAR expression in **Figures 4B–I**), which preceded and prevented later-stage aberrant bone bridging. However, from a perspective of gene expression quantity, quantitative reverse transcription polymerase chain reaction (RT-qPCR) analysis indicated that the expression levels of key mRNA markers associated with osteoclastogenesis, including CTSK, OSCAR, PTPN11, MAP3K7, and RELA, as well as the expression of three pro-inflammatory cytokines, were significantly downregulated in the GPI + SHP099 group compared to the GPI group (**Figures 4B–I**). Among the pro-inflammatory cytokines assessed, IL-6 and IL-1β demonstrated the most substantial reduction in expression levels following SHP099 treatment, suggesting a potent anti-inflammatory effect of SHP099 in the context of growth plate injury. These findings indicate that SHP099 effectively inhibits osteoclastogenesis and mitigates the inflammatory response associated with growth plate injury, potentially offering a novel therapeutic approach for managing post-injury complications.

### 3.3 IL-6 promotes phosphorylated SHP2 upregulation and osteoclastogenesis *in vitro*.

SHP2 is widely expressed in bone cell lineage, including osteoblasts and osteoclasts. To investigate osteoclast differentiation, we utilized a tartrate-resistant acid phosphatase (TRAP) staining assay to identify and characterize multinucleated osteoclasts. Osteoclasts were induced using recombinant RANKL (receptor activator of nuclear factor-kappa B ligand) derived from the 264.7 macrophage cell line, followed by TRAP staining (**Figure 3A**). Our observations indicated that the addition of the pro-inflammatory cytokine IL-6 significantly enhanced both the number of osteoclasts and their differentiation (**Figures 3A, B**), indicating increased osteoclast activity and function, which aligns with findings from previous studies (8, 21). Subsequently, we assessed the protein levels of SHP2, P65, TAK1, and their phosphorylated forms using Western blotting. The results demonstrated that IL-6 markedly upregulated the expression of phosphorylated SHP2 (p-SHP2) (**Figures 3C, D**). Moreover, the levels of SHP2, P65, phosphorylated P65 (p-P65), TAK1, and phosphorylated TAK1 (p-TAK1) exhibited an upward trend, although there were no statistically significant differences among the control, RANKL, and RANKL + IL-6 treatment groups (**Figures 3E–I**). To explore the secretion of pro-inflammatory cytokines, total RNA was extracted from treated cells, and the mRNA levels of IL-6, IL-1β, TNF-α, and osteoclast markers (CTSK and OSCAR) were measured via qPCR (**Figures 3J–N**). This analysis confirmed the successful induction of osteoclasts, revealing that all three cytokines could be produced and secreted by osteoclasts. Notably, IL-6 specifically stimulated an increase in the levels of IL-1β and its own expression. Furthermore, cell-free supernatants from the three groups were collected, and the concentrations of IL-6, IL-1β, and TNF-α were quantified using enzyme-linked immunosorbent assays (ELISA). The results showed a consistent trend among the three groups, corroborating the qPCR findings (**Figures 3O–Q**). In summary, our data indicate that IL-6 not only upregulates the protein expression of SHP2 and phosphorylated SHP2 (p-SHP2) but also establishes a positive feedback loop by promoting the secretion of IL-6 and IL-1β, both of which have been previously confirmed to enhance the formation and differentiation of osteoclasts.

### 3.4 SHP099 inhibits osteoclastogenesis targeting SHP2 *in vitro*

**Figure 5:**
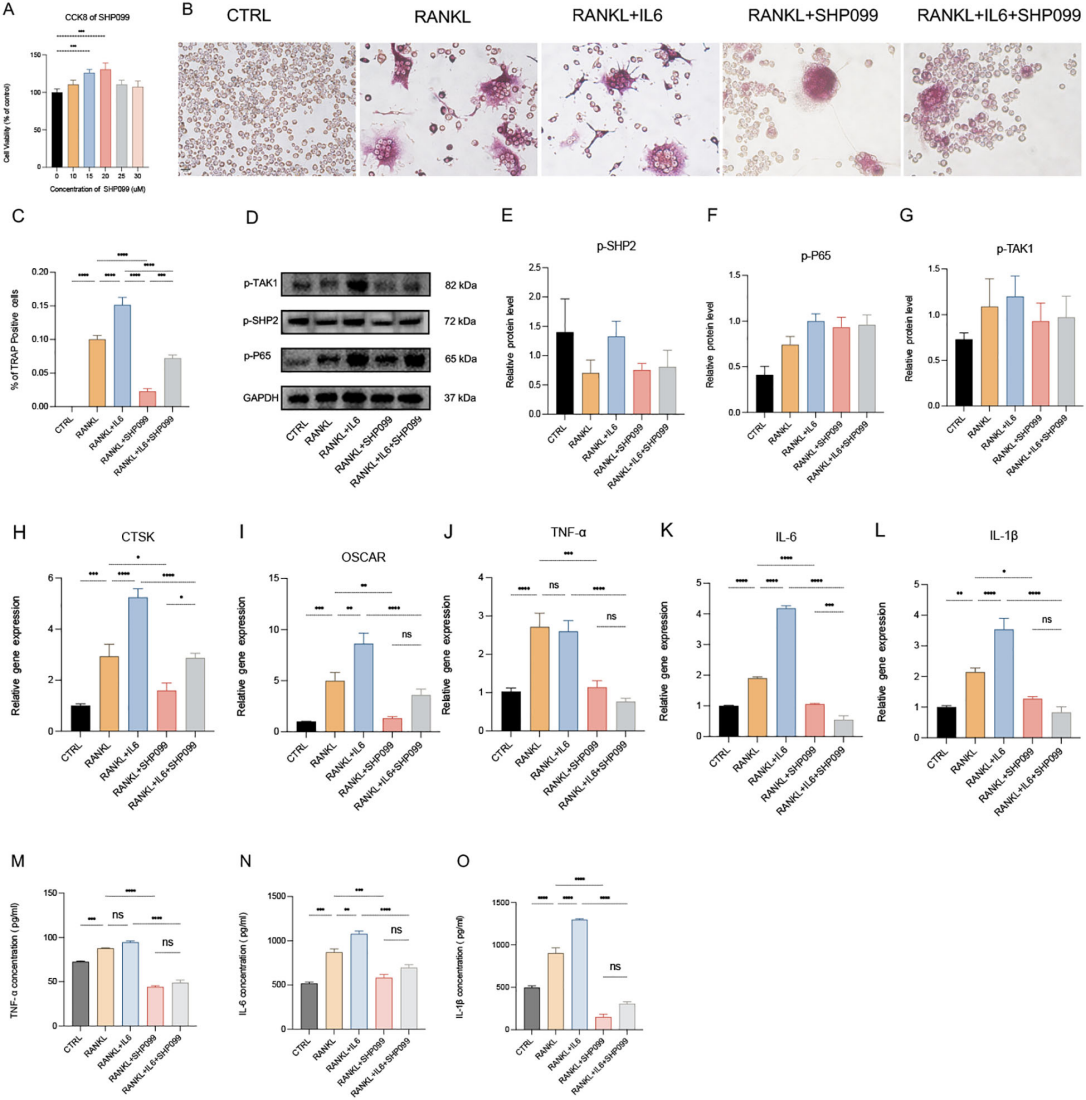
SHP099 inhibits the osteoclastogenesis targeting SHP2 *in vitro*. **(A)** The optimal drug concentration of SHP099 was measured by CCK-8 assay. **(B)** TRAP staining of RANKL-induced (100 ng/mL) osteoclasts treated with or without IL-6 (100 ng/mL) and/or SHP099 (15 μM) for 5 days. Multinucleated cells (≥3 nuclei) were quantified under light microscopy (40×). **(C)** Multinucleated osteoclastic cells were quantified by the amount of TRAP‐positive multinucleated cells (n=6). **(D)** Western blot analysis of phosphate SHP2 (p-SHP2), phosphate p65 (p-P65) and phosphate TAK1 (p-TAK1) in RAW264.7 after RANKL stimulation with or without IL-6 treatment for 5 days. **(E-G)** semiquantitative analysis of Western blot. (n ≥ 3). **(H-L)** qPCR analysis of mRNA of interleukin-1β (IL-1β), IL-6, tumor necrosis factor α (TNF-α), Cathepsin K (CTSK) and Osteoclast Associated Ig-Like Receptor (OSCAR). (n ≥ 6). **(M-O)** Enzyme-linked immunosorbent assay (ELISA) results of TNF-α, IL-1β and IL-6 secretion. (n=3). One-way ANOVA test. *P < 0.05, **P < 0.01, ***P < 0.001, ****P < 0.0001, ns represents no significance. All data are shown as the mean SEM.

SHP099 is a selective inhibitor of SHP2. To evaluate the effects of SHP099 on osteoclast differentiation *in vitro*, we first determined the optimal effective concentration of SHP099 to be 15 μM, using a concentration gradient from 0 μM to 25 μM as assessed by the CCK-8 assay (**Figure 5A**). Subsequently, we established five experimental groups: Control (CTRL), RANKL, RANKL + IL-6, RANKL + SHP099, and RANKL + IL-6 + SHP099. Equal volumes of cell suspension were seeded in 6-well culture plates, and the optimal concentrations of RANKL, IL-6, and SHP099 were added. The cultures were maintained for 5–6 days. Morphological assessment revealed that the number and size of osteoclasts in the RANKL + SHP099 and RANKL + IL-6 + SHP099 groups were significantly reduced compared to the RANKL and RANKL + IL-6 groups, respectively (**Figures 5B, C**). To further elucidate the molecular mechanisms underlying these observations, we analyzed the expression levels of phosphorylated proteins, specifically p-SHP2, p-TAK1, and p-P65, using Western blotting. The results demonstrated that the gray values of the protein bands for p-SHP2 and p-TAK1 in the RANKL + SHP099 and RANKL + IL-6 + SHP099 groups were markedly lower than those in the RANKL and RANKL + IL-6 groups, respectively (**Figure 5D**). Although the semiquantitative analysis indicated a consistent trend, no statistically significant differences were observed (**Figures 5E, G**). Moreover, we noted a significant reduction in the mRNA expression levels of osteoclast markers CTSK and OSCAR, further supporting the conclusion that SHP099 impairs both the quantity and quality of osteoclasts (**Figures 5H, I**). Interestingly, while p-P65 levels were not significantly suppressed by SHP099 treatment (**Figures 5D, F**), the relative gene expression analyses indicated that pro-inflammatory cytokines were notably lower in the SHP099-treated groups compared to those without SHP099, as measured by both PCR and ELISA assays (**Figures 5J–O**). Taken together, our findings demonstrate that SHP099, by specifically targeting SHP2, effectively inhibits the formation and differentiation of osteoclasts derived from RAW 264.7 cells *in vitro*, highlighting its potential as a therapeutic agent in osteoclast-mediated bone diseases.

### 3.5 Necessary role of TAK1\NF-KB signaling pathway in osteoclastogenesis

**Figure 6:**
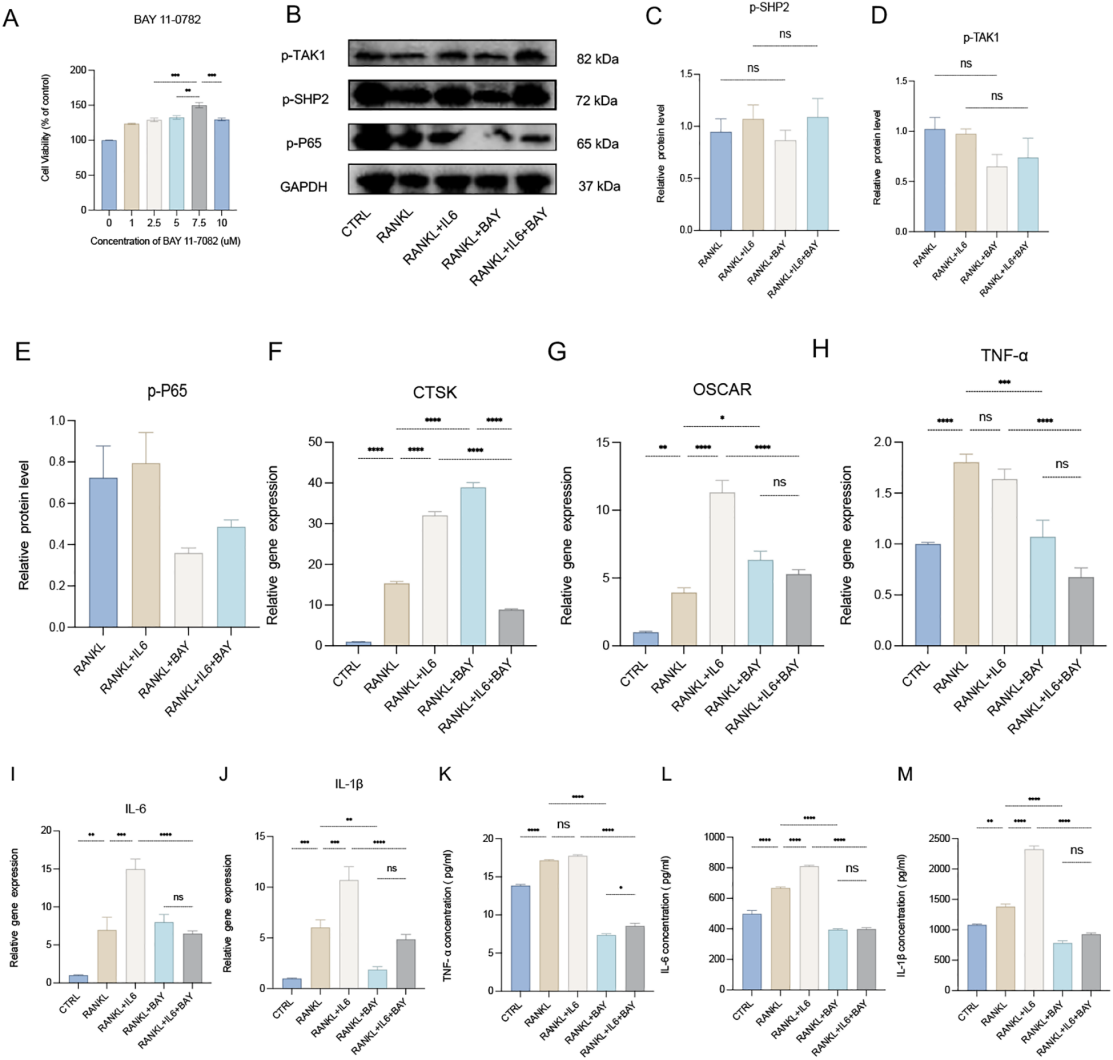
Necessary role of TAK1\NF-κB signaling pathway for osteoclastogenesis. **(A)** The optimal drug concentration of BAY 11–7082 was measured by CCK-8 assay. **(B)** Western blot analysis of phosphate SHP2 (p-SHP2), phosphate p65 (p-P65) and phosphate TAK1 (p-TAK1) in RAW264.7 after RANKL stimulation with or without IL-6 treatment for 5 days. **(C-E)** semiquantitative analysis of Western blot. (n ≥ 3). **(F-J)** qPCR analysis of mRNA of Cathepsin K (CTSK), Osteoclast Associated Ig-Like Receptor (OSCAR), interleukin-1β (IL-1β), IL-6 and tumor necrosis factor α (TNF-α). (n ≥ 6). **(K-M)** Enzyme-linked immunosorbent assay (ELISA) results of TNF-α, IL-1β and IL-6 secretion. (n=3). One-way ANOVA test. *P < 0.05, **P < 0.01, ***P < 0.001, ****P < 0.0001, ns represents no significance. All data are shown as the mean SEM. Note: Contrast with **Figures 5D-G** to appreciate SHP099’s upstream specificity versus BAY 11-7082’s downstream NF-κB inhibition.

The NF-κB signaling pathway plays a crucial role as a transcriptional factor in osteoclastogenesis. To explore this pathway’s involvement, we utilized BAY 11-7082, a specific inhibitor of the NF-κB subunit P65. The CCK-8 assay identified 7.5 μM as the optimal concentration of BAY 11-7082, effectively inhibiting the activity of P65 (**Figure 6A**). When RAW 264.7 cells were induced to differentiate into osteoclasts using RANKL, the addition of BAY 11-7082 (7.5 μM) significantly inhibited the augmented osteoclastogenesis induced by IL-6, as evidenced by the downregulation of osteoclast markers CTSK and OSCAR in the RANKL + IL-6 + BAY group (**Figures 6F, G**). This finding highlights the inhibitory effect of BAY on IL-6-mediated enhancement of osteoclastogenesis. Western blot analysis further revealed that the levels of phosphorylated SHP2 (p-SHP2) and phosphorylated TAK1 (p-TAK1) were not significantly altered following BAY treatment, while the expression level of phosphorylated P65 (p-P65) was notably reduced (**Figures 6B–E**). This suggests that while BAY effectively targets the NF-κB pathway, it does not directly affect the activation of SHP2 or TAK1 in this context. Additionally, qPCR and ELISA assays demonstrated that the production of pro-inflammatory cytokines, including IL-6, IL-1β, and TNF-α, was significantly decreased in the cells treated with BAY 11-7082 (**Figures 6H–M**). This reduction underscores the role of the NF-κB signaling pathway as a classical pro-inflammatory pathway essential for osteoclast differentiation and function. In conclusion, our findings indicate that the TAK1/NF-κB signaling pathway is critically necessary for osteoclastogenesis, with BAY 11–7082 effectively inhibiting the inflammatory signaling that promotes osteoclast differentiation.

## 4 Discussion

In summary, our study elucidates the critical role of SHP2 in osteoclastogenesis in response to IL-6 during the repair of injured growth plate cartilage. We demonstrated that SHP2 is prominently expressed at the injury site in a mouse tibial growth plate drill-hole model, with its activation being significantly influenced by IL-6 through phosphorylation processes. The selective SHP2 inhibitor SHP099 effectively attenuated osteoclast presence and reduced the expression of key osteoclast markers, including cathepsin K and OSCAR, alongside pro-inflammatory cytokines such as IL-6, IL-1β, and TNF-α. Furthermore, recombinant IL-6 was shown to enhance the expression of SHP2, TAK1, and the transcription factor NF-κB in osteoclasts derived from RAW 264.7 cells, underscoring the significance of the IL-6/SHP2 signaling pathway in promoting osteoclast differentiation. Notably, while SHP2 and TAK1 were negatively regulated by SHP099, the NF-κB pathway remained unaffected, indicating a complex interplay among these signaling molecules. The accompanying mechanism diagram (**Figure 2**) visually illustrates how IL-6 activates the osteoclastogenesis signaling pathway via SHP2, providing valuable insights into the relationship between inflammation and bone repair.

### 4.1 IL-6 enhances SHP2 phosphorylation and osteoclastogenesis

Our results clearly demonstrate that IL-6 significantly increases the phosphorylation of SHP2, thereby facilitating osteoclast differentiation. This finding aligns with existing literature that emphasizes the critical role of IL-6 in bone metabolism. IL-6 is known to activate multiple signaling pathways, including JAK/STAT and MAPK/ERK, which play pivotal roles in the differentiation of osteoclast precursors into mature osteoclasts (22–26). Specifically, IL-6 has been shown to upregulate the expression of RANK and its ligand RANKL, further stimulating osteoclastogenesis (8, 27–29). Our data not only corroborate these findings but also highlight the importance of SHP2 as a downstream mediator of IL-6 signaling in osteoclast development. This suggests that targeting IL-6 or its associated signaling pathways could offer a promising strategy for managing conditions characterized by excessive osteoclast activity, such as osteoporosis.

### 4.2 The inhibitory role of SHP099 on osteoclast differentiation

The application of SHP099 resulted in a marked inhibition of osteoclastogenesis, evidenced by the downregulation of CTSK and OSCAR expression. The role of SHP2 in osteoclast function has been well-documented, with studies indicating that SHP2 is essential for mediating the effects of RANKL on osteoclast precursor cells (11, 20, 30–33). Although we observed no significant changes in the levels of phosphorylated TAK1 and SHP2 following SHP099 treatment, the consistent trend of reduced osteoclast marker expression suggests that inhibiting SHP2 can effectively disrupt the signaling required for osteoclast differentiation. This is consistent with research that highlights the potential of SHP2 inhibitors as therapeutic agents in osteoclast-mediated diseases (15, 34–37). Thus, SHP099 may serve as a valuable tool in developing new treatments for disorders associated with excessive bone resorption.

### 4.3 BAY 11–7082 as a mechanistic comparator validating SHP099’s precision targeting

To contextualize SHP099’s mechanism, we employed BAY 11–7082 as a comparator. The critical role of the TAK1/NF-κB pathway in osteoclastogenesis was well-established, wherein NF-κB activation (e.g., p65 nuclear translocation) drove inflammatory osteoclast differentiation via RANK/RANKL induction (38–45). While BAY 11-7082 (a direct NF-κB inhibitor) confirmed this mechanism by suppressing IL-6-potentiated osteoclastogenesis (**Figure 6B**), its inclusion primarily served to delineate SHP099’s upstream specificity: SHP099 selectively inhibited the SHP2/TAK1 node (**Figure 5F**) without altering NF-κB activation (**Figures 6D, E**), whereas BAY 11–7082 blocked downstream p65 nuclear translocation (**Figure 6F**). This contrast demonstrated that SHP099’s anti-osteoclastogenic effect operated independently of broad NF-κB suppression, precisely intercepting the IL-6→SHP2→TAK1→NF-κB cascade at its upstream signaling components. Targeting SHP2/TAK1 (vs systemic NF-κB inhibition) preserved physiological NF-κB functions, reducing risks of immunosuppression while maintaining efficacy against pathological bone loss.

### 4.4 Inhibition of pro-inflammatory cytokine production

Further corroborating our findings, treatment with BAY 11–7082 resulted in a significant decrease in the production of pro-inflammatory cytokines, including IL-6, IL-1β, and TNF-α. These cytokines are well-known mediators of bone resorption and play critical roles in the crosstalk between osteoclasts and osteoblasts (27, 46). The reduction in these cytokines upon NF-κB inhibition indicates that the TAK1/NF-κB pathway is not only instrumental in osteoclast differentiation (47) but also in the broader context of inflammatory signaling in the bone microenvironment (48, 49). This suggests that pharmacological modulation of this pathway could potentially ameliorate the pathological processes leading to bone loss in inflammatory disorders.

### 4.5 Limitations and future directions

While this study elucidates the IL-6/SHP2/TAK1/NF-κB axis in osteoclastogenesis, several limitations warrant consideration: the murine growth plate injury model, while recapitulating core inflammatory pathways of pediatric fractures, exhibits interspecies differences: accelerated bone healing (murine 10 days ≈ human 4 weeks) and lack of age-dependent variations in growth plate composition seen clinically; RAW 264.7 cells, despite their utility, may not fully mirror *in vivo* osteoclast behavior due to immortalization artifacts; undetected off-target effects of SHP099 or BAY 11–7082 could confound mechanistic interpretations; exclusive focus on IL-6 overlooks potential synergism with other regulators (e.g., IL-17, OPG/RANKL). Future work should address these gaps by: (i) Validating SHP099 pharmacokinetics and long-term efficacy in skeletally immature large mammals (e.g., rabbits); (ii) Employing primary human osteoclast precursors and osteoblast-osteoclast co-cultures (e.g., MC3T3-E1) to assess cell-type-specific effects; (iii) Mapping cytokine interaction networks to identify combinatorial therapeutic targets.

## 5 Conclusion

Our findings provide significant insights into the mechanisms underlying osteoclastogenesis in the context of bone repair, emphasizing the therapeutic potential of targeting SHP2 to mitigate excessive bone resorption and prevent complications such as bone bridge formation following growth plate injuries. Future studies should explore the clinical applicability of SHP2 inhibitors in pediatric bone repair and their potential to enhance recovery outcomes in cases of growth plate injuries.

## Data Availability

The original datasets generated for this study are included in the article/**Supplementary Material**. Further inquiries can be directed to the corresponding authors.

## References

[1] Jones IE Williams SM Dow N Goulding A. How many children remain fracture-free during growth? a longitudinal study of children and adolescents participating in the Dunedin Multidisciplinary Health and Development Study. Osteoporos Int. (2002) 13:990–5. doi: 10.1007/s001980200137, PMID: 12459942

[2] Mizuta T Benson WM Foster BK Paterson DC Morris LL. Statistical analysis of the incidence of physeal injuries. J Pediatr Orthop. (1987) 7:518–23. doi: 10.1097/01241398-198709000-00003, PMID: 3497947

[3] Sananta P Lesmana A Alwy Sugiarto M. Growth plate injury in children: Review of literature on PubMed. J Public Health Res. (2022) 11:22799036221104155. doi: 10.1177/22799036221104155, PMID: 35923296 PMC9340334

[4] Chung R Foster BK Xian CJ. Injury responses and repair mechanisms of the injured growth plate. Front Biosci (Schol Ed). (2011) 3:117–25. doi: 10.2741/s137, PMID: 21196362

[5] Pichler K Musumeci G Vielgut I Martinelli E Sadoghi P Loreto C. Towards a better understanding of bone bridge formation in the growth plate - an immunohistochemical approach. Connect Tissue Res. (2013) 54:408–15. doi: 10.3109/03008207.2013.828715, PMID: 23941205

[6] Tong Z Yang X Li J. Research progress on the mechanism of interleukin-1beta on epiphyseal plate chondrocytes. Eur J Med Res. (2022) 27:313. doi: 10.1186/s40001-022-00893-8, PMID: 36575508 PMC9793524

[7] Mukkamalla SKR Malipeddi D. Myeloma bone disease: A comprehensive review. Int J Mol Sci. (2021) 22(12):6208. doi: 10.3390/ijms22126208, PMID: 34201396 PMC8227693

[8] Yokota K Sato K Miyazaki T Aizaki Y Tanaka S Sekikawa M. Characterization and function of tumor necrosis factor and interleukin-6-induced osteoclasts in rheumatoid arthritis. Arthritis Rheumatol. (2021) 73:1145–54. doi: 10.1002/art.41666, PMID: 33512089 PMC8361923

[9] Xiao W Shen Y Xu Y. LOX(G473A) induces the formation of osteoclasts in RAW264.7 cells via IL-6/JAK2/STAT3 signaling. Exp Cell Res. (2021) 409:112890. doi: 10.1016/j.yexcr.2021.112890, PMID: 34695437

[10] Shalev M Elson A. The roles of protein tyrosine phosphatases in bone-resorbing osteoclasts. Biochim Biophys Acta Mol Cell Res. (2019) 1866:114–23. doi: 10.1016/j.bbamcr.2018.07.005, PMID: 30026076

[11] Zhou Y Mohan A Moore DC Lin L Zhou FL Cao J. SHP2 regulates osteoclastogenesis by promoting preosteoclast fusion. FASEB J. (2015) 29:1635–45. doi: 10.1096/fj.14-260844, PMID: 25593124 PMC4415019

[12] Ishihara K Hirano T. Molecular basis of the cell specificity of cytokine action. Biochim Biophys Acta. (2002) 1592:281–96. doi: 10.1016/S0167-4889(02)00321-X, PMID: 12421672

[13] Jensen NR Kelly RR Kelly KD Khoo SK Sidles SJ LaRue AC. From stem to sternum: the role of Shp2 in the skeleton. Calcif Tissue Int. (2023) 112:403–21. doi: 10.1007/s00223-022-01042-3, PMID: 36422682

[14] Chen YN LaMarche MJ Chan HM Fekkes P Garcia-Fortanet J Acker MG. Allosteric inhibition of SHP2 phosphatase inhibits cancers driven by receptor tyrosine kinases. Nature. (2016) 535:148–52. doi: 10.1038/nature18621, PMID: 27362227

[15] Sun Z Liu Q Lv Z Li J Xu X Sun H. Targeting macrophagic SHP2 for ameliorating osteoarthritis via TLR signaling. Acta Pharm Sin B. (2022) 12:3073–84. doi: 10.1016/j.apsb.2022.02.010, PMID: 35865095 PMC9293663

[16] Sun H Patel N Ridwan SM Lottinger C Chen L Rowe D Kuhn L. Tricolor transgenic murine model for studying growth plate injury. J Vis Exp. (2024) 211:e66841. doi: 10.3791/66841, PMID: 39311607

[17] Bouxsein ML Boyd SK Christiansen BA Guldberg RE Jepsen KJ Müller R. Guidelines for assessment of bone microstructure in rodents using micro-computed tomography. J Bone Miner Res. (2010) 25:1468–86. doi: 10.1002/jbmr.141, PMID: 20533309

[18] Faul F Erdfelder E Lang AG Buchner A. G*Power 3: a flexible statistical power analysis program for the social, behavioral, and biomedical sciences. Behav Res Methods. (2007) 39:175–91. doi: 10.3758/BF03193146, PMID: 17695343

[19] Song C Yang X Lei Y Zhang Z Smith W Yan J. Evaluation of efficacy on RANKL induced osteoclast from RAW264.7 cells. J Cell Physiol. (2019) 234:11969–75. doi: 10.1002/jcp.27852, PMID: 30515780

[20] Wang L Yang H Huang J Pei S Wang L Feng JQ. Targeted Ptpn11 deletion in mice reveals the essential role of SHP2 in osteoblast differentiation and skeletal homeostasis. Bone Res. (2021) 9:6. doi: 10.1038/s41413-020-00129-7, PMID: 33500396 PMC7838289

[21] Wang T He C. TNF-α and IL-6: the link between immune and bone system. Curr Drug Targets. (2020) 21:213–27. doi: 10.2174/1389450120666190821161259, PMID: 31433756

[22] Hu L Liu R Zhang L. Advance in bone destruction participated by JAK/STAT in rheumatoid arthritis and therapeutic effect of JAK/STAT inhibitors. Int Immunopharmacol. (2022) 111:109095. doi: 10.1016/j.intimp.2022.109095, PMID: 35926270

[23] AiHaiti Y Song Cai Y Tuerhong X Ni Yang Y Ma Y Shi Zheng H. Therapeutic effects of naringin in rheumatoid arthritis: network pharmacology and experimental validation. Front Pharmacol. (2021) 12:672054. doi: 10.3389/fphar.2021.672054, PMID: 34054546 PMC8160516

[24] Blair HC Robinson LJ Zaidi M. Osteoclast signalling pathways. Biochem Biophys Res Commun. (2005) 328:728–38. doi: 10.1016/j.bbrc.2004.11.077, PMID: 15694407

[25] Dawalibi A Alosaimi AA Mohammad KS. Balancing the scales: the dual role of interleukins in bone metastatic microenvironments. Int J Mol Sci. (2024) 25(15):8163. doi: 10.3390/ijms25158163, PMID: 39125732 PMC11311339

[26] de Souza PPC Henning P Lerner UH. Stimulation of osteoclast formation by oncostatin M and the role of WNT16 as a negative feedback regulator. Int J Mol Sci. (2022) 23(6):3287. doi: 10.3390/ijms23063287, PMID: 35328707 PMC8953253

[27] Fischer V Haffner-Luntzer M. Interaction between bone and immune cells: Implications for postmenopausal osteoporosis. Semin Cell Dev Biol. (2022) 123:14–21. doi: 10.1016/j.semcdb.2021.05.014, PMID: 34024716

[28] Yoshimoto S Morita H Okamura K Hiraki A Hashimoto S. IL-6 plays a critical role in stromal fibroblast RANKL induction and consequent osteoclastogenesis in ameloblastoma progression. Lab Invest. (2023) 103:100023. doi: 10.1016/j.labinv.2022.100023, PMID: 36748192

[29] Wu Q Zhou X Huang D Ji Y Kang F. IL-6 enhances osteocyte-mediated osteoclastogenesis by promoting JAK2 and RANKL activity *in vitro* . Cell Physiol Biochem. (2017) 41:1360–9. doi: 10.1159/000465455, PMID: 28278513

[30] Dong H Liu X Duan J Zhang J Liu H Shen T. Excessive glucocorticoids combined with RANKL promote the differentiation of bone marrow macrophages (BMM) into osteoclasts and accelerate the progression of osteoporosis by activating the SYK/SHP2/NF-κB signaling pathway. Aging (Albany NY). (2024) 16:12263–76. doi: 10.18632/aging.206084, PMID: 39197167 PMC11424582

[31] Yang Y Yan Z Xie Q Wang Y Liu Z Lei M. Lactobacillus plantarum 45 activates SHP2 through inhibition of oxidative stress to regulate osteoblast and osteoclast differentiation. Aging (Albany NY). (2024) 16:6334–47. doi: 10.18632/aging.205708, PMID: 38575308 PMC11042941

[32] Ganesan R Rasool M. Interleukin 17 regulates SHP-2 and IL-17RA/STAT-3 dependent Cyr61, IL-23 and GM-CSF expression and RANKL mediated osteoclastogenesis by fibroblast-like synoviocytes in rheumatoid arthritis. Mol Immunol. (2017) 91:134–44. doi: 10.1016/j.molimm.2017.09.003, PMID: 28898718

[33] Bauler TJ Kamiya N Lapinski PE Langewisch E Mishina Y Wilkinson JE. Development of severe skeletal defects in induced SHP-2-deficient adult mice: a model of skeletal malformation in humans with SHP-2 mutations. Dis Model Mech. (2011) 4:228–39. doi: 10.1242/dmm.006130, PMID: 21068439 PMC3046097

[34] Zhu Y Wu Z Yan W Shao F Ke B Jiang X. Allosteric inhibition of SHP2 uncovers aberrant TLR7 trafficking in aggravating psoriasis. EMBO Mol Med. (2022) 14:e14455. doi: 10.15252/emmm.202114455, PMID: 34936223 PMC8899919

[35] Wang S Weng X Liu C Cheng B. SHP2 allosteric inhibitor SHP099 alleviates inflammation and restores salivary gland function in Sjögren’s disease-like animals via regulation of the IL-17RA signaling pathway. Int Immunopharmacol. (2025) 161:114986. doi: 10.1016/j.intimp.2025.114986, PMID: 40466613

[36] Pan J Qu J Fang W Zhao L Zheng W Zhai L. SHP2-triggered endothelial cell activation fuels estradiol-independent endometrial sterile inflammation. Adv Sci (Weinh). (2024) 11:e2403038. doi: 10.1002/advs.202403038, PMID: 39234819 PMC11538683

[37] Ying K Xin W Xu Y Lv D Zhu H Li Y. NanoSHP099-targeted SHP2 inhibition boosts Ly6C(low) monocytes/macrophages differentiation to accelerate thrombolysis. Adv Sci (Weinh). (2024) 11:e2308166. doi: 10.1002/advs.202308166, PMID: 38247197 PMC10987109

[38] Zheng X Qiu J Gao N Jiang T Li Z Zhang W. Paroxetine attenuates chondrocyte pyroptosis and inhibits osteoclast formation by inhibiting NF-κB pathway activation to delay osteoarthritis progression. Drug Des Devel Ther. (2023) 17:2383–99. doi: 10.2147/DDDT.S417598, PMID: 37605762 PMC10440089

[39] Zhao Y Wang C Qiu F Liu J Xie Y Lin Z. Trimethylamine-N-oxide promotes osteoclast differentiation and oxidative stress by activating NF-κB pathway. Aging (Albany NY). (2024) 16:9251–63. doi: 10.18632/aging.205869, PMID: 38809508 PMC11164488

[40] Xue C Luo H Wang L Deng Q Kui W Da W. Aconine attenuates osteoclast-mediated bone resorption and ferroptosis to improve osteoporosis via inhibiting NF-κB signaling. Front Endocrinol (Lausanne). (2023) 14:1234563. doi: 10.3389/fendo.2023.1234563, PMID: 38034017 PMC10682992

[41] Ouyang X Li S Ding Y Xin F Liu M. Foxf1 gene increases the risk of osteoporosis in rats by inhibiting osteoblast formation and promoting osteoclast differentiation through the upregulation of NF-κB pathway. J Musculoskelet Neuronal Interact. (2022) 22:242–50., PMID: 35642703 PMC9186464

[42] Mori K Mizokami A Sano T Mukai S Hiura F Ayukawa Y. RANKL elevation activates the NIK/NF-κB pathway, inducing obesity in ovariectomized mice. J Endocrinol. (2022) 254:27–36. doi: 10.1530/JOE-21-0424, PMID: 35638559

[43] Lin X Yuan G Yang B Xie C Zhou Z Liu Y. Dauricine attenuates ovariectomized-induced bone loss and RANKL-induced osteoclastogenesis via inhibiting ROS-mediated NF-κB and NFATc1 activity. Phytomedicine. (2024) 129:155559. doi: 10.1016/j.phymed.2024.155559, PMID: 38579642

[44] Tsubaki M Seki S Takeda T Chihara A Arai Y Morii Y et al. The HGF/Met/ NF-kB pathway regulates RANKL expression in osteoblasts and bone marrow stromal cells. Int J Mol Sci. (2020) 21(21):7905. doi: 10.3390/ijms21217905, PMID: 33114380 PMC7663721

[45] Cai P Yan S Lu Y Zhou X Wang X Wang M et al. Carnosol inhibits osteoclastogenesis *in vivo* and *in vitro* by blocking the RANKL−induced NF−kB signaling pathway. Mol Med Rep. (2025) 31(1):4. doi: 10.3892/mmr.2024.13369, PMID: 39422028 PMC11544531

[46] Hu K Shang Z Yang X Zhang Y Cao L. Macrophage polarization and the regulation of bone immunity in bone homeostasis. J Inflammation Res. (2023) 16:3563–80. doi: 10.2147/JIR.S423819, PMID: 37636272 PMC10460180

[47] Yang W Lu X Zhang T Han W Li J He W. TAZ inhibits osteoclastogenesis by attenuating TAK1/NF-κB signaling. Bone Res. (2021) 9:33. doi: 10.1038/s41413-021-00151-3, PMID: 34253712 PMC8275679

[48] Liu X Shen X Wang H Wang J Ren Y Zhang M. Mollugin prevents CLP-induced sepsis in mice by inhibiting TAK1-NF-κB/MAPKs pathways and activating Keap1-Nrf2 pathway in macrophages. Int Immunopharmacol. (2023) 125:111079. doi: 10.1016/j.intimp.2023.111079, PMID: 38149576

[49] Wang F Zhang W Wang C Fang X Cheng H Liu S. Inhibitor of Tec kinase, LFM-A13, decreases pro-inflammatory mediators production in LPS-stimulated RAW264.7 macrophages via NF-κB pathway. Oncotarget. (2017) 8:34099–110. doi: 10.18632/oncotarget.16212, PMID: 28415764 PMC5470954

