# Supplementary material for: SHP2 inhibition by SHP099 attenuates IL-6–driven osteoclastogenesis in growth plate injury
Source: Front Immunol. 2025 Aug 15;16:1659230. doi: 10.3389/fimmu.2025.1659230 (PMC12394037; doi:10.3389/fimmu.2025.1659230)
Supplement: Supplementary file 1 [file Table1.docx]

**Supplementary Table 1.** Reagents and antibody details.

| Reagents | Catalog numbers | Sources | Concentration |
| --- | --- | --- | --- |
| Recombinant Mouse TNFSF11/RANKL/CD254 Protein | RP02134 | ABclonal | 100 ng/ml |
| Mouse IL-6 Recombinant Protein | #216-16-2 | Peprotech | 100 ng/ml |
| BAY 11-7082 | #19542-67-7 | MCE | 7.5 μM |
| SHP099 | S6388 | Selleck | 15 μM |
| Anti-SHP2 antibody | AP0267 | ABclonal | 1:2000 |
| Anti-Phospho-SHP2 antibody | A12486 | ABclonal | 1:2000 |
| Anti-TAK1 antibody | F0413 | Selleck | 1:1000 |
| Anti-Phospho-TAK1 antibody | #9339 | CST | 1:2000 |
| Anti-NF-κB/p65 antibody | F0006 | Selleck | 1:1000 |
| Anti-Phospho-NF-κB/p65 antibody | F0155 | Selleck | 1:1000 |
| HRP Tag Rabbit Monoclonal Antibody | AG8064 | Beyotime | 1:2000-1:5000 |
| Mouse Gapdh qPCR Primer Pair | QM00014S | Beyotime | 1nmol each |
| Mouse Ctsk qPCR Primer Pair | QM11702S | Beyotime | 1nmol each |
| Mouse Oscar qPCR Primer Pair | QM60194S | Beyotime | 1nmol each |
| Mouse Rela qPCR Primer Pair | QM04918S | Beyotime | 1nmol each |
| Mouse Ptpn11 qPCR Primer Pair | QM04814S | Beyotime | 1nmol each |
| Mouse Map3k7 qPCR Primer Pair | QM06146S | Beyotime | 1nmol each |
| Mouse TNF-α qPCR Primer Pair | QM05522S | Beyotime | 1nmol each |
| Mouse IL-6 qPCR Primer Pair | QM03482S | Beyotime | 1nmol each |
| Mouse IL-1β qPCR Primer Pair | QM03422S | Beyotime | 1nmol each |
